# Report of Diseases in the Public and Private Practice of One of the Physicians of the Finsbury Dispensary, from the 20th of October to the 20th of November

**Published:** 1805-12

**Authors:** J. Reid

**Affiliations:** Grenville Street, Brunswick Square


*Report of Diseases in the public and private Practice of one of the Physicians of the Finsbury Dispensary, from the 20th of October to the 20th of November.*

| | | | |
|---|---|---|---|
| Diarrhœa | 15 | Morbi cutanei | 7 |
| Dysenteria | 2 | Morbi infantiles | 13 |
| Dyspepsia | 10 | Catarrhus | 18 |
| Hepatitis | 5 | Phthisis pulmonalis | 9 |
| Hydrops | 7 | Rheumatismus | 8 |
| Dyspnœa | 3 | Podagra | 1 |
| Amenorrhœa | 12 | Apoplexia | 5 |
| Chlorosis | 9 | Hysteria | 2 |
| Menorrhagia | 4 | Ephemera | 7 |
| Epilepsia | 1 | Scarlatina | 5 |
| Asthenia | 14 | | |

Coughs and derangements of the intestinal canal are the never failing epidemics of this particular season of the year.

In the history, and within the precincts of the Reporter's observation, they have never occurred so frequently; and what is worthy of remark, they have, for the most part, been accompanied by a pyrexial state, which, altho' not either in its essence or cause exactly the same as typhus, has approximated to the typhoid countenance and character, and of course, appeared to indicate a method of treatment considerably analogous to that which is required in the management of the latter disease.

Oppression of bodily strength and of mental power, have, for some weeks past, shewn themselves the prominent and nearly universal features of morbid affection.

The air, more especially in London, in November, has an apparent and important influence upon the faculties and feelings of our frame. The muscles are relaxed, the nerves, to make use of an intelligible phrase, although it is founded upon a false physiology, are *unstrung*, and the spirits in a greater or less degree depressed, according to the varied proportion of individual susceptibility to be acted upon by physical and exterior causes. Our bodies are constantly immersed in a bath of "volatile corruption," the obnoxious influence of which must especially be experienced by valetudinarians or other persons, who, after feasting during the summer and autumnal months upon upon the enlivening luxury of marine and rural oxygen, have recently returned to inhale, and be enveloped by the unwholesome and oppressive miasmata of the metropolis.

Scarlet fever has been of frequent occurrence; a disease once extremely formidable, but which has become much less so in consequence of modern amelioration in the theory and practice of medicine.

The cold, or rather tepid ablution, which latter, whilst perhaps attended with nearly all the advantages, is not accompanied with some of the risks and inconveniencies that are apt to ensue from the former, ought to be diurnally employed from the first day of the disease until the last of its continuance. When such treatment has been accurately adhered to, little fear may in general be entertained with regard to a favourable and satisfactory result. It may be right, however, to notice that in this disorder, a deficiency of general, is not unfrequently connected at the same time with an excess of local excitement, which is calculated to occasion some vacillation of judgment, and some uncertainty in the practice of the physician.*

During the long continued series of his reports, the author has not mentioned, except in his list, the class of dropsical affections, although no complaints have fallen more frequently under his observation, are accompanied with more distressing symptoms, or, are more generally fatal in their ultimate issue. The little impression that medicine is capable of producing upon such cases has perhaps, been one reason why they have not been particularly noticed. For the most part, they are, both in the inferior and higher classes of society, the melancholy result of protracted intemperance. The patient of either rank will in general be found, in spite of his indiscretion, to enjoy a freedom from positive indisposition, and an ordinary and comfortable degree of vigor, until a little after he has passed forty years of age. At that period, he is, for the first time, attacked with general dropsy, a dropsy of the abdomen, or a dropsy of the chest.

The debauchee is not aware that, although the ruin of his frame appears obvious and abrupt, the causes which ultimately

* "Any body may be a judge," said a young man to one holding that office, "who can distinguish between black and white." "You forget, my friend," replied the Judge, "that in law there are *grey* cases." There are many grey cases in medicine. *Moore's Medical Sketches.*

ultimately affected it, have been long and silent in their operation. There is no imprudence with regard to health that does not *tell*; and those are found in the event to suffer most essentially, that do not appear to suffer immediately from every individual act of indiscretion. It is such free livers, of robust and sturdy stamina, that are most liable to the distressing and almost invariably fatal disease, which is so faithfully as well as feelingly delineated by an author of the present day.

"It is often found impracticable even to relieve the dropsy of intemperance. The dropsical can have no reasonable expectation of being able to enjoy the pleasures of existence in full measure. In that dreadful complaint, dropsy of the chest or lungs, the fox-glove in particular, and sometimes other medicines, will often procure a respite, and the patient will seem to himself quite renovated; but the gleam is generally short; the tide flows back; the distress recommences; the same means, indeed, commonly procure another interval; but it is less perfect and shorter. At last it comes to be, as on board a ship on springing a leak that cannot be stopped; no sooner do the pumps cease to work than the water rises in the hold. If medicine discharges the water one day, it is collected in as great quantity in the next. The absorbents now soon begin to be insensible to the spur; then the horrors of slow suffocation commence, and a succession of spectacles are presented, at sight of which, the reflecting by-standers may well regret being endowed with animation, and may envy the very stones under their feet, for their insensibility."*

J. REID.

*Grenville Street, Brunswick Square.*

---

* Beddoes' Hygëia, Essay 8th.

MEDICAL

